# Effectiveness and prediction of treatment adherence to guided internet-based cognitive behavioral therapy for health anxiety: A cohort study in routine psychiatric care

**DOI:** 10.1016/j.invent.2024.100780

**Published:** 2024-10-16

**Authors:** Susanna Österman, Erland Axelsson, Erik Forsell, Cecilia Svanborg, Nils Lindefors, Erik Hedman-Lagerlöf, Volen Z. Ivanov

**Affiliations:** a Centre for Psychiatry Research, Department of Clinical Neuroscience, Karolinska Institutet, Region Stockholm, Sweden; b Division of Family Medicine and Primary Care, Department of Neurobiology, Care Sciences and Society, Karolinska Institutet, Stockholm, Sweden; c Liljeholmen University Primary Health Care Centre, Academic Primary Health Care Centre, Region Stockholm, Stockholm, Sweden; d Division of Psychology, Department of Clinical Neuroscience, Karolinska Institutet, Stockholm, Sweden; e Gustavsberg University Primary Health Care Centre, Academic Primary Health Care Centre, Region Stockholm, Stockholm, Sweden; f Stockholm Health Care Services, Region Stockholm, Sweden

**Keywords:** Internet-based cognitive- behavioural therapy, Health anxiety, Hypochondriasis, Routine care, Effectiveness study

## Abstract

**Objective:**

Health anxiety, also known as hypochondriasis, is a common psychiatric disorder which leads to considerable distress and is associated with high societal costs. Internet-based cognitive behavioural therapy (ICBT) for health anxiety has demonstrated efficacy in randomized controlled trials (RCTs), but there is limited knowledge regarding its effectiveness in real-world settings. This study aimed to evaluate the clinical effectiveness of guided ICBT for health anxiety in routine psychiatric care, including symptom change, treatment adherence, and potential negative effects. Additionally, we explored predictors of treatment adherence.

**Method:**

A longitudinal cohort study of 447 patients enrolled in 12 weeks of ICBT for health anxiety between 2018 and 2020 in an outpatient psychiatric clinic specializing in ICBT. Primary outcome measure was the 14-item Short Health Anxiety Inventory (SHAI-14) and a within-group design with repeated measures was utilized for the primary analysis.

**Results:**

Participants showed significant improvements from pre- to post-treatment (*d* = 1.61). At post-treatment, 60 % (95 % CI 58–62) demonstrated statistically reliable change (RCI), and 44 % (95 % CI 42–46) were in remission. On average, participants completed 7 (SD = 4) out of 12 treatment modules. For each additional completed module, the mean reduction was 0.31 (95 % CI 0.10 to 0.54) points on the SHAI-14.

**Conclusions:**

Guided ICBT for health anxiety can be effective when delivered within the context of routine psychiatric care. The study suggests that effect sizes are comparable with those in RCTs and higher treatment adherence is associated with better outcomes in health anxiety. ICBT could be used to increase availability to effective therapy for health anxiety.

## Highlights

• Evaluated guided ICBT for adult patients with health anxiety in an outpatient clinic
• Reductions in symptoms of health anxiety and a large effect size at post-treatment
• Adherence to treatment was associated with better outcomes.
• No serious adverse events were reported.

## 1 Introduction

Health anxiety is primarily characterized by a recurrent and excessive fear of, or preoccupation with, having a serious disease. Patients with health anxiety, also known as hypochondriasis, typically receive a diagnosis of somatic symptom disorder or illness anxiety disorder according to the DSM-5 (Association AP, 2013; World Health Organization, 1992). The point-prevalence of the condition has been estimated to 3.4 % in the general population (Sunderland et al., 2013), but health anxiety problems seem to affect up to 20 % of patients in medical clinics (Tyrer et al., 2011). Health anxiety leads to distress and functional impairment, diminished quality of life and a heightened risk of further psychiatric conditions (Barsky et al., 1998; Creed and Barsky, 2004) and risk of premature death (Mataix-Cols et al., 2023). Beyond the personal impact, health anxiety incurs substantial societal costs and burden on the healthcare system (Barsky et al., 2001).

Health anxiety can be effectively treated with cognitive-behavioural therapy (CBT) (Olatunji et al., 2014) but a major challenge in the dissemination of CBT is the limited access to trained therapists (Cavanagh, 2014). Internet-based CBT (ICBT) is a promising format that requires less therapist time (Hedman et al., 2012). The treatment content is provided through text, video, or audio via a secure digital platform, and can be delivered with or without guidance from a clinician. A core principle of ICBT is that the patient works with the same components as in conventional CBT.

The efficacy of ICBT for health anxiety has been supported by findings from several randomized controlled trials (RCTs) (Hedman et al., 2011; Hedman et al., 2014; Hedman et al., 2016; Newby et al., 2018), but there is limited knowledge of its effectiveness in routine psychiatric care, i.e., under real-world conditions (Porzsolt et al., 2015). It is important to study the benefits and the potential negative effects in routine care and during clinical implementation as the generalizability of results from efficacy studies can be limited due to several factors including highly structured diagnostic and treatment procedures and restrictive eligibility criteria (Andersson et al., 2019; Rozental et al., 2014; Shadish et al., 2000). Two recent studies from the same clinic, have highlighted the potential of ICBT in treating health anxiety, demonstrating large within-group effect sizes on the primary outcome (d = 0.89 to 1.66) within a routine clinical setting (Newby et al., 2020; Sharrock et al., 2021). However, these studies did not employ initial diagnostic assessments, leaving questions about the participants' actual diagnoses of health anxiety and any comorbid conditions unanswered. Moreover, the majority of participants (73.8 %) (Newby et al., 2020) were enrolled in unguided ICBT, meaning that data on the efficacy of clinician-guided ICBT in regular psychiatric care remains scarce.

One factor of importance in moving from RCTs to routine clinical use is adherence: the extent to which patients engage with the treatment (van Ballegooijen et al., 2014). It is commonly noted that adherence can be lower in routine care settings, likely affecting effectiveness (Donkin et al., 2011; Hilvert-Bruce et al., 2012). Understanding the factors that influence adherence is important, as they can guide efforts to improve patient engagement and treatment outcomes. A recent study examining two RCTs of ICBT for health anxiety (Axelsson and Hedman-Lagerlöf, 2023) found that being male, having lower educational attainment, and perceiving the treatment as less credible were associated with lower treatment adherence. The question of which factors predict treatment adherence in routine care however remains unanswered.

Additionally, assessing potential negative outcomes in psychological treatments, such as a worsening of the symptoms initially targeted for treatment (deterioration) or the emergence of new, unwanted symptoms as a consequence of the treatment, is essential (Rozental et al., 2018). Understanding these aspects can help refine intervention strategies to better manage or mitigate adverse effects, ensuring safer and more effective treatment delivery in clinical settings.

### 1.1 Aim of the study

The aim of this study was to evaluate the effectiveness of clinician-guided ICBT for health anxiety within routine psychiatric care in Sweden. This included assessing symptom change from pre- to post-treatment, treatment adherence, patient satisfaction, and potential negative effects such as deterioration and side effects. Additionally, we explored predictors of adherence to treatment. We hypothesized that patients would make significant improvements on both measures of health anxiety and depressive symptoms and that the level of adherence would be in line with previous effectiveness research on ICBT (40–50 % completed modules) (Newby et al., 2020; Andersson and Hedman, 2013; Etzelmueller et al., 2020).

## 2 Material and methods

### 2.1 Design and setting

This was a longitudinal cohort study of ICBT for health anxiety (N = 447) at an ICBT outpatient clinic, the Internet Psychiatry Clinic in Stockholm, Sweden. This clinic is publicly funded and serves adults across Sweden with ICBT-treatments for a range of psychiatric disorders (Hedman et al., 2013a). The study was approved by the Regional Ethics Board of Stockholm (2017/2320-32). All patients were informed about the study and consented to their data being used for research. No one opted out; thus, all participant data were included. The study was preregistered at ClinicalTrials.gov (NCT04921280) and reported in accordance with STROBE guidelines for observational studies (von Elm et al., 2007).

### 2.2 Recruitment and sample

We used data from patients enrolled between April 2018 (when the treatment was introduced) and December 2020 (when data was extracted due to the clinic's transition to a new treatment platform). All patients were self-referred by submitting an online application with a comprehensive battery of self-report instruments. A structured diagnostic assessment was then conducted at the clinic or via video conference by a psychiatrist, a resident physician, supervised by a psychiatrist or clinical psychologist. According to the clinic's guidelines, patients should (a) have a principal diagnosis of DSM-5 illness anxiety disorder or somatic symptom disorder with a pronounced fear of serious disease; (b) be 16 or older; (c) not have hindering psychiatric comorbidities like ongoing substance abuse, a psychotic disorder, or moderate to high suicide risk; and (d) not have severe concentration, reading/writing/language difficulties, or a serious somatic illness (in cases of ADHD, individual assessments were made regarding the ability to engage with treatment material) The Health Preoccupation Diagnostic Interview (HPDI) (Axelsson et al., 2016) assessed illness anxiety disorder and somatic symptom disorder according to DSM-5 (American Psychiatric Association, 2000) while the Mini International Neuropsychiatric Interview (MINI) (Lecrubier et al., 1998) assessed other psychiatric disorders. Severity of the primary disorder was rated using the Clinical Global Impressions Severity Scale (CGI-S) (Attkisson and Zwick, 1982), see eTable 1 in the online supplement. Patients not suitable for ICBT or needing alternative treatments were referred to appropriate care. During the study, 709 individuals applied and 447 (63.0 %) started treatment. Table 1 provides an overview of the included patients.

**Table 1:** Characteristics of patients enrolled in ICBT for health anxiety.

Variable	Patients with health anxiety (N = 447)
Age, M (SD), min–max	36 (11), 16–81
Women, *n* %	305 (66.9 %)
**Time since debut of first HA symptoms, *n* (%)**	
<1 years	41 (9.2 %)
1–5 years	99 (22.2 %)
6–10 years	82 (18.3 %)
>10 years	216 (48.3 %)
Unknown	9 (2.0 %)
Previous CBT treatment (any), *n* (%)	175 (39.2 %)
**Occupational status, *n* (%)**	
Working	355 (79.4 %)
Employed with workplace accommodations	2 (0.5 %)
Student	67 (15.0 %)
Unemployed	11 (2.5 %)
Sick leave	2 (0.5 %)
Retired	10 (2.2 %)
**Education, *n* (%)**	
7–9 years in school	4 (0.9 %)
Incomplete vocational or secondary school	20 (4.5 %)
Vocational school	16 (3.6 %)
Secondary school	80 (17.9 %)
University, uncompleted studies	64 (14.3 %)
University, completed studies^a^	263 (58.8 %)
Married or de facto	332 (74.3 %)
**Current medications, *n* (%)**	
Antidepressant	47 (10.5 %)
Anti-anxiety medication	52 (11.6 %)
Sleep Medication	28 (6.3 %)
**Psychiatric comorbidities, *n* (%)**	
Depressive disorder	70 (15.7 %)
Generalized anxiety disorder	61 (13.7 %)
Panic disorder	59 (13.3 %)
Social anxiety disorder	27 (6.1 %)
Agoraphobia	27 (6.1 %)
Insomnia	27 (6.1 %)
ADHD	7 (1.6 %)
OCD	3 (0.7 %)
Exhaustion disorder	2 (0.5 %)
History of at least one suicide attempt, *n* (%)	11 (2.5 %)
History of inpatient psychiatric care, *n* (%)	14 (3.1 %)

*Note*. Abbreviations: HA; health anxiety, CBT; cognitive behavioural therapy, ADHD; attention deficit hyperactivity disorder, OCD; Obsessive-compulsive disorder.

a Completed university studies were self-reported by participants and defined as having completed any university degree (including university college, bachelor's, or master's degrees).

### 2.3 Outcome measures

#### 2.3.1 Health anxiety

The primary outcome was the 14-item Short Health Anxiety Inventory, SHAI-14 (Salkovskis et al., 2002). Each item is scored 0–3, resulting in a total score range of 0–42. The SHAI-14 has good internal consistency, construct validity, sensitivity to change, and 1-week test-retest reliability (*r* = 0.78) (Alberts et al., 2013; Te Poel et al., 2017). Scores of 0–27 indicate no to mild health anxiety, 28–32 moderate health anxiety, and 33–42 substantial health anxiety (Österman et al., 2022). Cronbach's alpha in this sample ranged between 0.88 and 0.94.

#### 2.3.2 Depressive symptom

Depressive symptoms were measured using the 9-item Montgomery Åsberg Depression Scale-Self rated (MADRS-S) (Svanborg and Åsberg, 1994) with a total score range of 0–54. The instrument has shown satisfactory 1-week test-retest reliability (ICC = 0.78) and is sensitive to change (Fantino and Moore, 2009). Cronbach's alpha in this sample ranged between 0.85 and 0.91.

#### 2.3.3 General anxiety symptoms

The 7-item Generalized Anxiety Disorder Scale (GAD-7) (Spitzer et al., 2006) was used to measure general anxiety symptoms, with a total score range of 0–21. The GAD-7 demonstrates good construct validity, sensitivity to change (Beard and Björgvinsson, 2014), and 1-week test-retest reliability (ICC = 0.83) (Spitzer et al., 2006). Cronbach's alpha in this sample ranged between 0.87 and 0.89.

#### 2.3.4 Patient satisfaction and treatment credibility

Patient satisfaction was measured with the 8-item Client Satisfaction Questionnaire (CSQ-8) (Attkisson and Zwick, 1982) ranging from 8 to 32, with higher scores indicating greater satisfaction. Cronbach's alpha in this sample was 0.90. Treatment credibility and expectancy were assessed using the 5-item Credibility/Expectancy Questionnaire (CEQ) (Devilly and Borkovec, 2000) ranging from 0 to 50, with higher scores indicating higher perceived credibility. Cronbach's alpha in this sample was 0.88.

#### 2.3.5 Adherence and adverse events

Adherence was assessed based on the number of modules (out of a total of 12) completed by patients at post-treatment (Hadjistavropoulos et al., 2016). At post-treatment, we assessed the occurrence of adverse events by asking the patients to report whether they had experienced any negative or unwanted effects due to the treatment. If answering ‘Yes’, patients were asked to specify those effects in free text.

### 2.4 Assessment points

All self-report questionnaires were administered online, a valid format according to previous research (Hedman et al., 2015a), on the same platform as the treatment. Health anxiety and depressive symptoms were assessed during screening (3–4 weeks before treatment), pre-treatment, weekly during the 12-week treatment, post-treatment, and at a 6-month follow-up (completed by 29 % of patients; see eTable 2 in the online supplement). General anxiety symptoms were assessed at pre-treatment, post-treatment, and 6-month follow-up. Treatment credibility was measured at week two, and adherence, treatment satisfaction, and negative events were measured post-treatment.

### 2.5 Treatment program

The ICBT program, developed by Hedman et al. (Hedman et al., 2011; Axelsson et al., 2020), consisted of 12 online modules with self-help texts, worksheets, and audio files for mindfulness practice. Modules covered psychoeducation, exposure with response prevention, and relapse prevention (see Table 2). The treatment content and process have been detailed previously (Axelsson et al., 2020). Each module ended with homework assignments and questions, followed by individualized feedback from a licenced psychologist, who then provided access to the next module. The clinic's team consisted of 14 clinical psychologists specialized in ICBT, and the same psychologist usually communicated with the patient throughout the treatment via asynchronous text messages. Therapist support was tailored to the participant's needs, typically focusing on monitoring the patient's progress, encouraging engagement with the treatment material, and addressing any questions. Patients could generally expect up to 3 messages per week from their therapist, with at least one message per week as a minimum, though this could vary. Therapist time spent writing messages and reviewing homework was automatically recorded by the platform itself throughout the entire treatment period. This included both time spent on the platform working with each participant and the number of messages written by the therapist and patient. The core treatment lasted 12 weeks, with module completion pace determined by the patient. An additional week allowed patients to ask final questions, ensuring clarity and closure. After completion, patients retained access to the platform and materials for six months, without therapeutic support.

**Table 2:** Treatment content.

Module	Content and purpose
1	Psychoeducation about health anxiety. Introduction to CBT.
2	Review of the CBT model for health anxiety. Introduction to mindfulness to handle bodily symptoms and to enable exposure exercises.
3	Psychoeducation about fear of bodily symptoms. Interoceptive exposure aimed at evoking conditioned fear responses to bodily sensations.
4	Systematic response prevention based on the patients' behavioural diaries.
5	Exposure to real-world situations (in vivo) and activities that may trigger health anxiety.
6	Imaginal exposure through writing an “illness story” about the patients' most feared outcomes.
7	Continued imaginal exposure. Additional exposure exercises to address the fear of death.
8	Common obstacles to successful exposure and how to overcome these. Continued exposure and response prevention.
9	Continued exposure and response prevention.
10	Continued exposure and response prevention.
11	Treatment summary. Introduction to values to increase life quality.
12	Relapse prevention and how to handle health-care utilization in the future.

*Note.* Abbreviations: CBT; Cognitive Behavioural Therapy.

### 2.6 Statistical analyses

Statistical analyses were conducted using Stata/SE 15.1 and R version 4.2.0 (Team, 2013) including the MICE package (Van Buuren and Groothuis-Oudshoorn, 2011) on an intention-to-treat basis, assuming data were missing at random. For the primary outcome measure SHAI-14 and MADRS-S, we used linear mixed effects models with a random intercept and slope to analyse changes from pre-treatment to post-treatment, focusing on the fixed effect of time. This model was selected because it appropriately handles missing data for repeated continuous measures by using all available data points. A similar model, but with only a random intercept, was used for GAD-7, which was only administered pre- and post-treatment.

A per protocol analysis of the primary outcome was also conducted including participants who completed at least six modules, based on the assumption that such participants had been exposed to all components of the ICBT program (Hedman et al., 2011). To assess the impact of treatment adherence on health anxiety over time, we extended the mixed-effects model described above, adding adherence (number of completed modules) as a simple effect and in interaction with time. Additionally, we tested for a curvilinear interaction between adherence and time by including a quadratic term.

Logistic regression was used to examine predictors of adherence (completing at least six modules), and included age, gender (female: 1, male: 0), post-secondary education (yes: 1, no: 0), SHAI-14 and MADRS-S scores at pre-treatment, therapy-naïve status (yes: 1, no: 0) and treatment credibility ratings. Standardized within-group effect sizes (Cohen's d) were calculated as the difference between pre- and post-treatment means divided by the pre-treatment standard deviation (Cohen, 1988; Becker, 1988). Alpha for all analyses was set at 0.05.

Response and remission rates were calculated on imputed data using multiple imputation by chained equations with the MICE package (Van Buuren and Groothuis-Oudshoorn, 2011) in R. This method was chosen as it allows us to leverage the entire dataset, including auxiliary variables, to generate more plausible imputed values. Imputation was carried out with five imputations and five iterations using predictive mean matching. Responders were defined and reported in multiple ways: as having either a 30 % or 50 % reduction on the SHAI-14 or using the Reliable Change Index (RCI) of 8 points. The RCI was calculated based on a test-retest reliability of *r* = 0.78 (Te Poel et al., 2017) using the formula 1.96 ∗ SD ∗ √2 ∗ √(1-test-retest reliability) where the standard deviation (SD) was 5.5 (Salkovskis et al., 2002). Remission was defined as scoring below the SHAI-14 cut-off of 18 (Österman et al., 2022). Deterioration was defined as an RCI exceeding 0.84, equating to a change of at least 4 points on the SHAI-14, as suggested by Wise (Wise, 2004). This method, using an 80 % one-sided test (rather than a 97.5 % one-sided test), was chosen to detect even small deteriorations, minimizing the risk of underestimating negative effects, despite potential ceiling effects and regression to the mean from baseline.

## 3 Results

### 3.1 Attrition and treatment adherence

In total, 447 patients (100 %) completed the pre-treatment assessment, and 321 (71.8 %) completed the post-treatment assessment (see eTable 2 for missing data per week). On average, patients completed 7.3 out of 12 modules (SD = 3.6), with 292 participants (65 %) completing at least 6 modules, classified as “completers” (see eTable 3 for detailed completion rates). Patients sent a mean of 14.6 messages (SD = 8.8) to therapists, who spent an average of 12.3 min (SD = 11.4) per patient per week.

### 3.2 Change in self-reported symptoms

**Fig. 1:**
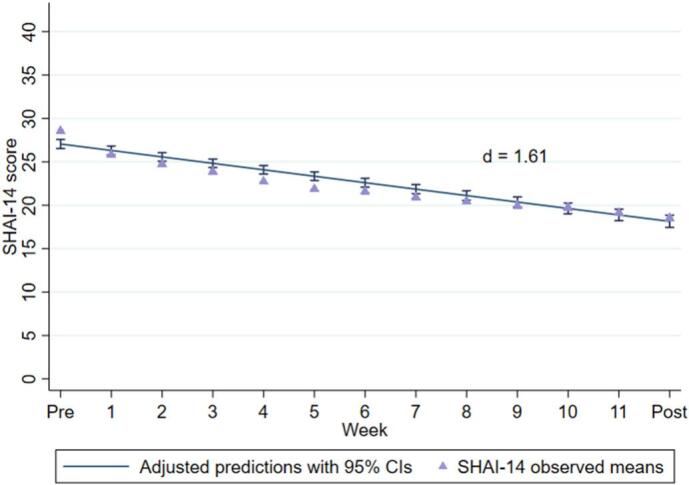
Observed and estimated mean SHAI-14 scores over time (pre, weekly, post). Error bars represent 95 % confidence intervals. Abbreviations: SHAI-14, the 14-item Short Health Anxiety Inventory; d, Cohen's d within-group effect size, CI; Confidence interval.

Means, SDs and effect sizes for the symptom measures are presented in Table 3. The mixed-effects model showed a significant decrease in health anxiety symptoms over 12 weeks on the primary outcome SHAI-14 (mean reduction = −8.99, (95 % CI -9.67 to −8.30), (Fig. 1) with a large within-group effect size between pre- and post-treatment (*d* = 1.61). At post-treatment, 195 (43.6 %, [95 % CI 41.8 to 45.5 %]) participants had a score below 18 on the SHAI-14 indicating remission. Table 4 shows responder rates on the primary outcome based on the different definitions used. As shown in Table 3, patients were also improved on measures of depression and general anxiety. The per protocol analysis indicated a large decrease in health anxiety symptoms over time on the SHAI-14 with a mean reduction of −9.26 (95 % CI -10.03 to −8.50; *d* = 1.74). The number of participants providing data for the 6-month follow-up assessment was 129 (29 %). With a 71 % data loss, a 6-month ITT follow-up analysis could not be performed. For the complete case analysis, see eTable 2 in the online supplement.

**Table 3:** Model estimates for all measures from baseline to the primary endpoint (post-treatment) from the linear mixed effects models (Intention-to-treat, N = 447).

Measure	M (SE)	Within group difference (95 % CI)	Within-group Cohen's d
SHAI-14			
Pre	27.10 (0.27)		
Post	18.10 (0.35)	−8.99 (−8.30 to −9.67)	1.61
MADRS-S			
Pre	13.31 (0.33)		
Post	7.45 (0.35)	−5.86 (−5.19 to −6.53)	0.72
GAD-7			
Pre	9.87 (0.24)	−2.45 (−1.63 to −3.28)	0.54
Post	7.42 (0.35)		

*Note.* Abbreviations: M; Mean, SE Standard error; SHAI-14, the 14-item Short Health Anxiety Inventory; MADRS-S, the Montgomery-Åsberg Depression Rating Scale - Self-rated; GAD-7, the Generalized Anxiety Disorder Scale.

**Table 4:** Secondary outcomes of ICBT for health anxiety in routine care: clinical improvement, adherence, credibility, satisfaction, and adverse events summary (N = 447).

Variable	Outcome
Adherence	M (SD)
No. completed modules,	7.3 (3.6)
No. participants completing ≥6 modules, %, (95 % CI)	292, 65.1 %


Variable	Outcome
Dichotomous outcomes based on the SHAI-14	n, %, (95 % CI)
Remission	195, 43.6 % (41.8–45.5)
Response RCI 8 points	270, 60.3 % (58.4–62.1)
Response 30 % reduction	253, 56.6 % (54.7–58.5)
Response 50 % reduction	99, 22.1 % (20.6–23.7)
Deterioration RCI 4 points	10, 2.2 % (1.7–2.8)
Deterioration 30 % increase	7, 1.5 % (1.1–2.0)


Variable	Outcome
Credibility and satisfaction	M (SD)
CEQ	36.35 (8.09)^a^
CSQ-8	25.45 (4.10)^b^
Adverse events (at least one) n % (95 % CI)	64, 20.1 % (16.1–24.9)^b^

*Note.* Remission is defined as a score below 18 points at post-treatment on the SHAI-14. Response is defined as a statistically reliable change (RCI) of 8 points or more on the SHAI-14) or percentual change on the SHAI-14 (30 & 50 %). Deterioration is defined as a RCI of 4 points on the SHAI-14. ^a^ CEQ was measured at week 2, n = 428. ^b^ CSQ-8 and adverse events were measured at post-treatment, n = 321. Abbreviations: M; Mean, SD; Standard deviation, SHAI-14; the 14-item Short Health Anxiety Inventory, CEQ; Credibility/Expectancy Questionnaire, CSQ-8; Client Satisfaction Questionnaire.

### 3.3 Impact of treatment adherence

**Fig. 2:**
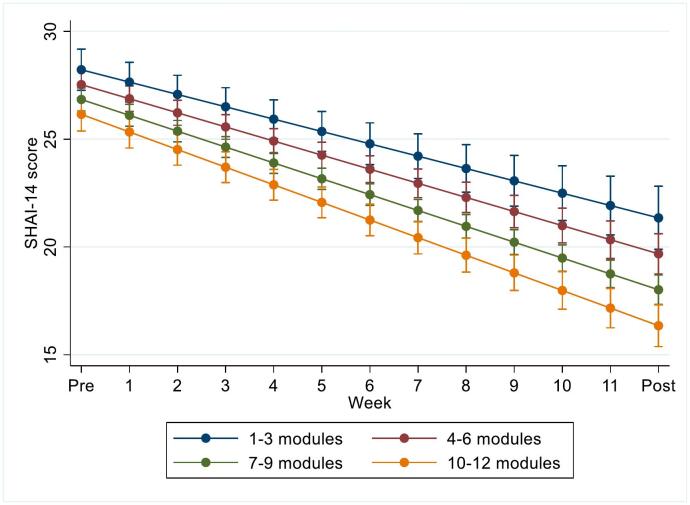
Estimated SHAI-14 mean scores over time (pre, weekly, post) with 95 % CIs, as a function of numbers of modules completed (out of 12), based on a linear mixed effects model. Abbreviations: SHAI-14, the 14-item Short Health Anxiety Inventory, CI; Confidence interval.

There was a significant interaction between module completion and time on health anxiety symptoms (*p* = 0.004). For each additional module completed, participants' SHAI-14 scores improved by an additional average of 0.3 (95 % CI 0.1 to 0.5) from pre- to post-treatment. Based on the same linear mixed effects model, the estimated mean improvement on the SHAI-14 for a patient who completed all 12 modules was 10.0 (95 CI% 9.0 to 11.1) and for 6 modules, it was 8.1 (95 % CI 7.3 to 9.0) (see Fig. 2). There was no significant curvilinear effect of the number of modules (adherence × adherence × time), indicating no difference in symptom improvement between completing 1 to 2, 5 to 6, or 10 to 11 modules. For detailed results on predictors of adherence, see Table 5.

**Table 5:** Potential predictors of adherence in ICBT for health anxiety.

Predictors	OR (95 % CI)
Sociodemographic predictors	
Age (years)	1.03 (1.01 to 1.05)⁎
Female	1.16 (0.77 to 1.75)
Post-secondary education	1.72 (1.12 to 2.64)⁎
Clinical predictors	
Health anxiety (SHAI-14)	0.978 (0.95 to 1.01)
Depression (MADRS-S)	0.975 (0.95 to 1.00)
Therapy-naive (psychological)	0.47 (0.29 to 0.76)⁎
Credibility/expectancy (CEQ)	1.10 (1.07 to 1.13)⁎

Prediction of adherence in terms of completion of ≥6 modules. ^a^ORs derived from analyses of continuous predictors stand for the proportion of odds as a function of a one-unit increase in the predictor. Abbreviations: ICBT: Internet-based cognitive-behavioural therapy; SHAI-14: 14-item Health Anxiety Inventory; MADRS-S: Montgomery-Åsberg Depression Rating Scale – Self-report version; CEQ: Credibility/Expectancy scale; OR: odds ratio.

⁎ P < 0.05.

### 3.4 Treatment credibility, patient satisfaction, and adverse events

The mean CSQ-8 score at post-treatment was 25.5 (SD = 4.1) out of 32, indicating high patient satisfaction (see eTable 4). For example, 295 (91.9 %) answered that they “would most likely/probably recommend the treatment to a friend”. For credibility ratings, see Table 4 and eTable 5. Of the 321 patients (71.8 %) who provided post-measures, 64 (19.9 %) reported at least 1 adverse event, with no serious events requiring hospitalization. The most reported events were increased but transient anxiety from working with exposure (48.4 %) and stress related to the treatment format (20.3 %). Symptom deterioration was rare (see Table 4).

## 4 Discussion

### 4.1 Principal findings

In this cohort study of 447 patients, we evaluated the effectiveness of guided ICBT for health anxiety within routine psychiatric care. Our study showed significant reductions in health anxiety symptoms alongside notable improvements in depressive symptoms in the patients. Providing this treatment only required therapists to spend around 14 min per week with each patient. Moreover, patients reported high satisfaction with the treatment and considered the treatment format to be credible. Adherence to the program was moderate, and our findings revealed a significant association between treatment module completion and reductions in SHAI-14 scores. This suggests that higher treatment adherence may lead to better outcomes in health anxiety.

### 4.2 Comparison with previous research

The treatment effect on health anxiety symptoms (d = 1.61) observed in our study is comparable with those from previous research on ICBT for health anxiety. Specifically, our results are consistent with those reported in a prior RCT using the same program (Hedman et al., 2016), for both health anxiety (d = 1.55) and depression (d = 0.42). Furthermore, our study serves as a particularly relevant comparison to the effectiveness trial by Newby et al., (Newby et al., 2020) which reported large within-group effect sizes for ICBT (1.66). The improvements we observed also align with the outcome from a meta-analysis of many trials of CBT for health anxiety (Axelsson and Hedman-Lagerlöf, 2019) which demonstrated substantial within-group effect sizes for the reduction of health anxiety (g = 1.76). This meta-analysis also reported a pooled remission rate of 48 % after CBT, regardless of format, which is similar to the 43.6 % estimated in our study. Overall, our findings suggest that the positive effects of ICBT for health anxiety are generalizable beyond RCT settings to routine psychiatric care.

### 4.3 Treatment adherence

Our findings on the significant association between adherence and symptom reduction align with previous studies (Hilvert-Bruce et al., 2012; Hedman et al., 2015b; Hedman et al., 2013b). As expected, adherence in this study, defined as an average completion rate of 7.3 out of 12 modules; 60.7 %, was somewhat lower than in a previous RCT of the same treatment (8.6 out of 12 modules, 71.7 %) (Hedman et al., 2016). The adherence were also marginally lower than those in the effectiveness trial conducted by Newby and colleagues (Newby et al., 2020), which reported an average completion rate of 4.2 out of 6 modules (70.5 %) in the guided treatment. This difference was not expected, especially given the similarities in the guided format of the ICBT programs. This finding should be interpreted cautiously, as the discrepancy may be attributable to differences in the number of modules, treatment content, participant demographics or other unknown factors. We found that higher educational attainment, higher age, experience of prior psychological treatment, and perceiving the treatment as more credible were significant predictors of treatment completion (defined as finishing at least 6 modules). Overall, our results were similar to previous research (Axelsson and Hedman-Lagerlöf, 2023), with the exception that we did not find an association with gender, and that having experience of psychological therapy was as a significant predictor. Reasons for adherence are likely multifaceted, but these results suggest that several background factors may influence the likelihood of patients completing ICBT. Regardless, the adherence rates in this study highlight the challenges of engagement and adherence in ICBT (Etzelmueller et al., 2020) and underscore the need for strategies to enhance patient motivation. Potential strategies could include refining the treatment content (Beatty and Binnion, 2016), optimizing the user interface (Lemon et al., 2020; Hentati et al., 2021), and implementing systems to identify and support patients at risk for treatment failure with tailored interventions (Forsell et al., 2019). Future studies with more rigid experimental designs are needed to uncover the causality behind the predictive role of module completion for ICBT outcomes.

### 4.4 Negative events and deterioration

Regarding safety, few participants, 2.2 % (CI 1.7–2.8 %), demonstrated symptom deterioration, while 20.1 % (CI 16.1–24.9 %) reported at least one negative event, mainly transient anxiety and unpleasant feelings. This aligns with a recent meta-analysis of CBT and ICBT for health anxiety (Axelsson and Hedman-Lagerlöf, 2023). The second most common complaint was stress related to the treatment format, such as content scope and time commitment, in line with previous studies on the negative effects of ICBT (Rozental et al., 2015) highlighting the need to refine ICBT programs. Potential enhancements include streamlining content, adding interactive elements, and offering flexible pacing to meet individual needs.

### 4.5 Strengths and limitations

A key limitation of this study was the lack of a control group, raising the possibility that within-group effects were not caused by the ICBT, including both beneficial and unwanted outcomes. However, ICBT's efficacy for health anxiety has been demonstrated in RCTs, with many benefits here exceeding typical spontaneous recovery in control groups (g = 0.17) (Scott et al., 2022). Despite this, the study's strength lies in its ecological validity, reflecting ICBT's real-world effectiveness. Additionally, we studied a large sample, conducted thorough diagnostic assessments, and used psychometrically sound outcome measures, supporting the validity of our findings.

The generalizability of our findings may be restricted by the demographic composition of our sample which consisted of predominantly self-referred patients, mostly female (66.9 %), employed (79.4 %), with a university degree (58.8 %). These demographics might not be representative for individuals with more severe health anxiety. However, all patients in our study were diagnosed with illness anxiety disorder or somatic symptom disorder, indicating significant clinical distress and nearly half (48.3 %) had had symptoms of health anxiety for over a decade. Finally, the naturalistic setting led to a higher rate of post-treatment data loss than in previous RCTs which prevents conclusions about long-term treatment effectiveness.

### 4.6 Conclusions

This study provides support for the effectiveness of guided ICBT for health anxiety in a routine psychiatric setting, with marked improvements in symptoms of health anxiety and depression in large patient cohort. Treatment adherence appears to be an important factor for achieving positive outcomes.

## Funding

This work was supported by Bror Gadelius foundation. The funder had no role in the design and conduct of the study.

## Declaration of competing interest

The authors declare the following financial interests/personal relationships which may be considered as potential competing interests:

Dr Axelsson and Professor Hedman-Lagerlöf receive royalties from Natur & Kultur for a self-help book on health anxiety.

Other authors have no competing interests to declare.

## Appendix A Supplementary data

Supplementary material Image 1
