# Supplementary material for: Effectiveness and prediction of treatment adherence to guided internet-based cognitive behavioral therapy for health anxiety: A cohort study in routine psychiatric care
Source: Internet Interv. 2024 Oct 16;38:100780. doi: 10.1016/j.invent.2024.100780 (PMC11533681; doi:10.1016/j.invent.2024.100780)
Supplement: Supplementary file 1 — Supplementary material [file mmc1.docx]

Supplemental Online Content

Self-report measurements – examples of items

eTable 1. CGI-ratings of Patients at Pre-Treatment Assessment

eTable 2. Means, Standard Deviations, T-test Results, Number of Patients at Each Measurement Point, and Within-group Effect Sizes for Complete Case Analysis

eTable 3. Module-by Module Completion Rates for Patients

eTable 4. Treatment Satisfaction Ratings from 318 Patients at Post-Treatment: Frequencies and Percentages

eTable 5. Ratings of treatment credibility. Observed means and standard deviations from N=428 participants

**Self-report measurements – examples of items**

**Health anxiety.** The primary outcome was measured using the 14-item Short Health Anxiety Inventory (SHAI-14) (1). In this questionnaire, patients were instructed to read each group of statements and select the one that best described their feelings over the past week.

Example item:

(a) I do not worry about my health.

(b) I occasionally worry about my health.

(c) I spend much of my time worrying about my health.

(d) I spend most of my time worrying about my health.

**Depressive symptoms.** Depressive symptoms were measured using the 9-item Montgomery-Åsberg Depression Rating Scale-Self Rated (MADRS-S) (2). Patients were instructed to assess how they had been feeling during the past 3 days. The questionnaire consists of items where scales and statements describe various degrees of distress, ranging from none at all (0) to maximum (3).

Example item: Mood

(0) I can be either cheerful or sad, depending on the circumstances.

(1) I feel a bit low for the most part, though sometimes it eases up a little.

(2) I feel thoroughly low and gloomy. Even things that normally cheer me up give me no pleasure.

(3) I feel so utterly low and miserable that I can imagine nothing worse.

**General anxiety symptoms.** General anxiety symptoms were measured using the 7-item Generalized Anxiety Disorder Scale (GAD-7)(3). Patients were asked to state how often, during the past 2 weeks, they were bothered by each symptom. Response options were “not at all” (0), “several days” (1), “more than half the days” (2), and “nearly every day” (3).

Example item:

"Over the last 2 weeks, how often have you been bothered by the following problem?

Feeling nervous, anxious, or on edge?”

eTable 1. CGI-ratings of Patients at Pre-Treatment Assessment

| **CGI-S, N (%)** |  |
| --- | --- |
|  |  |
| CGI-S=2 | 23 (5.2%) |
| CGI-S=3 | 199 (44.5 %) |
| CGI-S=4 | 168 (37.6%) |
| CGI-S=5 | 42 (9.4%) |
| CGI-S=6 | 5 (1.1%) |
| Information unknown | 10 (2.2%) |

*Note*. Total *N* = 447. Abbreviation: CGI; Clinical Global Impressions-Severity Scale.

eTable 2. Means, Standard Deviations, T-test Results, Number of Patients at Each Measurement Point, and Within-group Effect Sizes for Complete Case Analysis

| **Outcome** | **Mean and (SD)** | **Cronbach's alpha** | **T-test** | **P-value** | **Cohens’ D** |
| --- | --- | --- | --- | --- | --- |
|  |  |  |  |  |  |
| **SHAI-14** |  |  |  |  |  |
| Pre (n=447) | 28.53 (5.60) | 0.88 |  |  |  |
| Week 1 (n=398) | 25.82 (6.15) | 0.91 |  |  |  |
| Week 2 (n=388) | 24.76 (6.32) | 0.91 |  |  |  |
| Week 3 (n=380) | 23.87 (6.37) | 0.92 |  |  |  |
| Week 4 (n= 359) | 22.72 (6.34) | 0.92 |  |  |  |
| Week 5 (n=333) | 21.86 (6.37) | 0.92 |  |  |  |
| Week 6 (n=344) | 21.59 (6.35) | 0.92 |  |  |  |
| Week 7 (n=319) | 20.92 (6.41) | 0.92 |  |  |  |
| Week 8 (n=310) | 20.45 (6.74) | 0.93 |  |  |  |
| Week 9 (n=286) | 19.90 (6.38) | 0.92 |  |  |  |
| Week 10 (n=271) | 19.73 (6.53) | 0.93 |  |  |  |
| Week 11 (n=263) | 19.14 (6.88) | 0.94 |  |  |  |
| Post (n=321) | 18.50 (6.39) | 0.93 | t = 24.85 | <.000 | -1.79 [-2.54, -1.03] |
| Follow-up (n=129) | 15.78 (7.06) | 0.94 | t = 18.29 | <.000 | 2.43 [ 3.70, -1.16] |
|  |  |  |  |  |  |
| **MADRS-S** |  |  |  |  |  |
| Pre (n= 447) | 14.48 (7.38) | 0.85 |  |  |  |
| Week 1 (n= 398) | 12.94 (7.14) | 0.87 |  |  |  |
| Week 2 (n= 386) | 11.62 (7.10) | 0.88 |  |  |  |
| Week 3 (n=378) | 11.07 (6.997) | 0.88 |  |  |  |
| Week 4 (n=358) | 9.896 (6.81) | 0.89 |  |  |  |
| Week 5 (n= 333) | 9.66 (6.73) | 0.89 |  |  |  |
| Week 6 (n=343) | 9.50 (7.01) | 0.90 |  |  |  |
| Week 7 (n= 318) | 9.11 (6.72) | 0.89 |  |  |  |
| Week 8 (n=310) | 8.95 (7.20) | 0.91 |  |  |  |
| Week 9 (n= 286) | 8.37 (6.40) | 0.89 |  |  |  |
| Week 10 (n= 271) | 7.85 (6.69) | 0.90 |  |  |  |
| Week 11 (n=263) | 7.79 (6.75) | 0.90 |  |  |  |
| Post (n=321) | 7.29 (6.61) | 0.91 | 17.24 | < .000 | -.90 [-1.70, -.14] |
| Follow-up (n= 129) | 6.57 (5.55) | 0.86 | 11.56 | < .000 | -.98 [-2.17, .22] |
|  |  |  |  |  |  |
| **GAD-7** |  |  |  |  |  |
| Pre (n=447) | 9.87 (4.93) | 0.87 |  |  |  |
| Post (n=282) | 6.19 (4.38) | 0.89 | 10.92 | <.000 | -.66 [-1.23, -.09] |
| Follow-up (n=129) | 4.54 (3.99) | 0.89 | 9.33 | < .000 | -.90 [-1.80, -.00] |

*Note*. Paired t-test results for all symptom measures from pre-treatment to post-treatment and 6-month follow-up. Abbreviations: M; Mean, SD; Standard deviation, SHAI-14;14-item Health Anxiety Inventory, MADRS-S; Montgomery-Åsberg Depression Rating Scale – Self-report version, GAD-7; the Generalized Anxiety Disorder Scale.

eTable 3. Module-by Module Completion Rates for Patients

| **Module completion** | **N** | **%** |
| --- | --- | --- |
| 1 | 31 | 6.94 |
| 2 | 26 | 5.82 |
| 3 | 32 | 7.16 |
| 4 | 32 | 7.16 |
| 5 | 34 | 7.61 |
| 6 | 36 | 8.05 |
| 7 | 36 | 8.05 |
| 8 | 31 | 6.94 |
| 9 | 30 | 6.71 |
| 10 | 33 | 7.38 |
| 11 | 21 | 4.70 |
| 12 | 105 | 23.49 |

eTable 4. Treatment Satisfaction Ratings from 318 Patients at Post-Treatment: Frequencies and Percentages

| **Question** | **No, absolutely not**  **n (%)** | **No, I do not think so**  **n (%)** | **Yes, I think so**  **n (%)** | **Yes, absolutely**  **n (%)** |
| --- | --- | --- | --- | --- |
| How would you rate the quality of the service you have received | 3 (0.9%) | 46 (14.5%) | 194 (61.0%) | 75 (23.6%) |
| Did you get the kind of service you wanted? | 3 (0.9%) | 51 (16.0%) | 201 (63.2%) | 63 (19.8%) |
| To what extent did the program meet your needs? | 3 (0.9%) | 73 (23.0%) | 178 (56.0%) | 64 (20.1%) |
| If a friend were in need of similar help, would you recommend the program to him or her? | 2 (0.6%) | 21 (6.6%) | 136 (42.8%) | 159 (50.0%) |
| How satisfied are you with the amount of help you have received? | 3 (0.9%) | 26 (8.2%) | 179 (56.3%) | 110 (34.6%) |
| Have the services you received help you deal more effectively with your problems? | 3 (0.9%) | 23 (7.2%) | 166 (52.2%) | 126 (39.6%) |
| In an overall general sense, how satisfied are you with the services you have received? | 2 (0.6%) | 38 (12.0%) | 180 (56.6%) | 98 (30.8%) |
| If you were to seek help again, would you come back to the service? | 3 (0.9%) | 45 (14.2%) | 137 (43.1%) | 133 (41.8%) |

*Note.* Treatment satisfaction was rated with the Client Satisfaction Questionnaire (CSQ-8).

eTable 5. Ratings of treatment credibility. Observed means and standard deviations from N=428 participants

| **Question** | **Mean (SD)** |
| --- | --- |
| How logical does the treatment seem? | 7.90 (2.03) |
| How sure are you that this method will be successful in helping with your health anxiety? | 6.34 (2.03) |
| How sure are you that you would recommend this kind of treatment to a friend with similar problems? | 7.48 (2.12) |
| How effective do you think this kind of treatment would be for another fear or worry? | 7.46 (1.88) |
| How much do you think you will improve from this treatment? | 7.17 (1.84) |

*Note.* Ratings of credibility/expectancy were done by patients at week two in treatment using the Credibility/Expectancy Questionnaire (CEQ).

**References:**

1. Salkovskis PM, Rimes KA, Warwick HMC, Clark DM. The Health Anxiety Inventory: development and validation of scales for the measurement of health anxiety and hypochondriasis. Psychological medicine. 2002;32(5):843-53.

2. Svanborg P, Åsberg M. A new self‐rating scale for depression and anxiety states based on the Comprehensive Psychopathological Rating Scale. Acta Psychiatrica Scandinavica. 1994;89(1):21-8.

3. Spitzer RL, Kroenke K, Williams JBW, Löwe B. A Brief Measure for Assessing Generalized Anxiety Disorder: The GAD-7. Archives of Internal Medicine. 2006;166(10):1092-7.
